# The aryl hydrocarbon receptor in T cells contributes to sustaining oral tolerance against ovalbumin in a mouse model

**DOI:** 10.17179/excli2017-168

**Published:** 2017-03-20

**Authors:** Daniel Biljes, Christiane Hammerschmidt-Kamper, Katja Merches, Charlotte Esser

**Affiliations:** 1 IUF - Leibniz Research Institute for Environmental Medicine, Auf'm Hennekamp 50, D-40225 Düsseldorf

**Keywords:** OT, aryl hydrocarbon receptor, T cells, mouse, mucosal immunology

## Abstract

Oral tolerance (OT) towards antigens encountered in the gut is a vital immune function of gut immunity. Experimental models can demonstrate OT efficacy by feeding of a protein followed by peripheral immunization and measuring the specific antibody titer. We had previously shown that exposure to 2,3,7,8-tetrachlorodibenzo-p-dioxin (TCDD), a xenobiotic high-affinity aryl hydrocarbon receptor (AhR)-ligand, destabilized OT against ovalbumin (OVA) in mice. AhR is involved in the development, differentiation and function of immune cells, and highly expressed in gut epithelial cells and gut immune cells. We here used AhR-deficient mice to study the role of AhR in OT further. We show that complete AhR-deficiency undermines the stability of oral tolerance against OVA upon multiple immunizations, despite no renewed oral encounter with the antigen. This OT destabilization is accompanied by significant changes in IL10 and TGFβ RNA in the gut tissue. Using conditional AhR-deficient mouse lines, we identify T cells as the major responsible immune cell type in this context. Our findings add to knowledge that lack of AhR signaling in the gut impairs important gut immune functions.

## Introduction

The immune system is vital to fight off pathogens and cancer cells. Dendritic cells (DC) and other antigen-presenting cells take up and proteins, present them as peptides on their cell surface in order to guide and instruct T cells, which recognize peptide antigens by their specific T cell receptor. An important and unique feature of the immune system is the distinction between (i) foreign and autoantigens, and (ii) between harmless and harmful antigens (Matzinger, 1994[32]; Pradeu and Cooper, 2012[38]). While all proteins are potential antigens, immune responses are normally not raised against one´s own body proteins, and in general only against pathogens. The underlying mechanisms are very complex and under intense investigation, as failure to make this distinction, and thus not to become tolerant - using the immunological term - against harmless antigens can cause debilitating autoimmune diseases or allergies, sometimes even death. Immunologic memory ensures that tolerance, once established, remains sustained, even during long stretches of not encountering again the original tolerizing antigen (Siewert et al., 2008[40]; Pabst and Mowat, 2012[36]; Cheroutre and Madakamutil, 2005[8]). 

The aryl hydrocarbon receptor (AhR), an evolutionary old ligand-activated transcription factor, is highly expressed in the intestinal epithelium and in many immune cells. Much evidence in the last years delineated a critical role for AhR in balancing inflammatory versus regulatory immune responses, e.g., by shifting T cell differentiation towards regulatory T cells, or instructing DC towards a tolerance-inducing phenotype. AhR links the environment to the immune system. Indeed, AhR may have evolved (or maybe hijacked during evolution) to be part of the immune system´s strategies to moderate immune responses by using natural or endogenous indoles, tryptophan derivatives, glucosinolates and other small molecular weight chemicals (Hubbard et al., 2016[21]; Soshilov and Denison, 2014[41]; Bessede et al., 2014[1]; Denison et al., 2011[10]). Genetic ablation of AhR in mice affects gut immunity, e.g., mice become more susceptible to *Citrobacter rodentium *infection, their gut microbiome changes, and the mice have a higher bacterial load in general (Li et al., 2011[28]; Kiss et al., 2011[26]; Murray et al., 2016[34]). Evidence regarding the skin and the gut suggests strongly that a certain amount of AhR-induction by dietary ligands is necessary for health; certain plants can provide such ligands (Haas et al., 2016[17]; Esser and Rannug, 2015[11]; Haarmann-Stemmann et al., 2015[16]). Oral tolerance (OT) is the term for the vital immunological phenomenon that no immune responses are made against antigens encountered via the oral route. OT has been described many decades ago. In mice, it is usually modeled by feeding of protein antigens (such as ovalbumin (OVA) or keyhole limpet hemocyanin), followed by immunization with the antigen (Weiner et al., 2011[44]; Pabst and Mowat, 2012[36]). Subsequently, either formation of antibodies is measured in the serum, or the capacity for T cell restimulation detected in vitro. It is still under debate whether antigen dose directs the underlying mechanisms such as T cell anergy and/or the increase of regulatory T cells (Treg). The main mechanisms of OT, starting with antigen sampling from the gut by CD103+DC, Treg generation in mesenteric lymph nodes, and homing of Treg back to the lamina propria have been clarified. OT against food antigens is both local and systemic, and must be distinguished from local tolerance against gut residing commensal bacteria. Food allergies and celiac disease are thought to result from impaired OT induction (Burks et al., 2008[4]; Makharia, 2014[31]) 

We had previously shown that persistent activation of the AhR by the high-affinity xenobiotic ligand 2,3,7,8-tetrachlorodibenzo-p-dioxin (TCDD) can break OT. Thus, TCDD-treated mice produced antibodies against the model antigen OVA after several boosting immunizations, despite previous tolerization (Chmill et al., 2010[9]). Our data in this study had suggested a DC-driven shift in the Treg/Th17 balance. To clarify the role of AhR in OT further, we here analyze AhR-deficient mice for stabile OT. Our data show that AhR is not critical for the establishment of OT, but contributes to its stability. AhR-expressing adaptive T cells are a major responsible cell population, indicating a role of AhR in establishing memory for OT. 

## Material and Methods

### Mice 

We used AhR-deficient mice (Schmidt et al., 1996[39]) or conditional AhR^∆lck^, AhR^∆CD11c^, and AhR^∆villin^ mice, which we bred by crossing AhR^flox/flox ^mice (Walisser et al., 2005[43]) with mice, which expressed Cre-recombinase under the respective promoter (CD11c-Cre (Caton et al., 2007[6]), Villin-Cre (Madison et al., 2002[30]), lck-Cre (Hennet et al., 1995[19])). Mice were tested for successful conditional deletion. We sorted target cells (for instance the intestinal epithelial cells for AhR^∆villin^) and various organ tissues, which do not contain the target cells, such as liver, thymus, B cells etc.) and analyzed for the* ahr *deletion by PCR. As known from the original publication, the CD11c-Cre mice deleted the *ahr* gene also in a minor fraction of T cells. In AhR^∆lck^, *ahr* was not efficiently deleted in gut γδ T cells, and consequently, these mice had the same frequency of γδ T cells than wild-type littermates. All other T cells deleted *ahr* in this line. For all mouse lines we used the line-specific wild-type littermates as controls. Mice were bred in our specific-pathogen free animal facility; mice were under a 12 hour/12 hour light-dark cycle, and had access to food and water ad libitum.

All experiments were done after obtaining permission of the relevant governmental bodies, in accordance with relevant German animal welfare laws. 

### OT induction 

For tolerization, mice were gavaged with 20 mg OVA (grade V;#A5503, Sigma-Aldrich, Munich, Germany) in PBS/20 g body weight on day 0, day 2, and day 5 of the experiment. Mice were then immunized i.p. day 12 with 10 µg OVA in Complete Freund´s adjuvant (CFA) and boosted on days 26, 33 and 40 with 10 µgOVA/20g body weight, dissolved in incomplete Freund´s adjuvant (IFA). In contrast to other protocols with low and medium dosage (e.g. 20 mg once or 0.5 mg OVA five times), which we tested in preliminary experiments, this high-dose tolerization protocol induced a stable tolerance upon several re-challenges. Serum samples were taken on the days of immunization. 

### OVA-ELISA

OVA-specific IgG1 antibodies in mouse serum were determined by ELISA. 96-well plates were coated with 100 µg/ml OVA overnight at 4 °C. Serum samples were titrated onto the plates and detected with goat anti-mouse IgG1 antiserum (Southern Biotech, Birmingham, USA) coupled to biotin. The ELISA was developed with avidin-HRP/TMB and values measured at 450 nm after addition of 0.75 µl H_2_SO_4_. All values are expressed relative to a standard from pooled sera of C57BL/6 mice immunized and boosted with OVA/IFA. 

### Flow cytometry of intraepitheliallymphocytes and lamina propria cells 

For isolation of immune cells from the intestinal epithelial cells and the lamina propria, the small intestine was resected, washed and cut into small pieces. For IEL, the pieces were placed in a PBS/10 % FCS/2mM EDTA solution for 30 min at 37° C in a shaking water bath. The solution was passed through a strainer and stored on ice for immediate staining. For isolation of lamina propria cells the epithelial-depleted residual intestinal tissue was placed for 20 minutes in a solution containing 0.15 mg/ml collagenase-P and 0.025 mg/ml DNAse-I (Roche, Mannheim, Germany). The solution was vigorously vortexed and then filtered through a 100 µm strainer. Lymphocytes/ leucocytes were then isolated on an EasyColl™ density gradient (Biochrome, Berlin, Germany), and immediately stained with fluorescently labeled antibodies. Fc receptors were blocked with CD16/32 (BioLegend, Fell, Germany). Dead cells were discriminated using Fixable Viability Dye eFluor 506 (eBioscience, Frankfurt/Main, Germany). Cells were analyzed in list-mode on a FACSCanto-II™ flow cytometer (Becton-Dickinson, Heidelberg, Germany). Live lymphocytes/leucocytes were gated according to scatter characteristics. Antibodies were: MHC-II (M5/ 114.15.2 BD-Biosciences, Heidelberg, Germany), CD11c (N418), CD103 (2E7, eBioscience, Frankfurt, Germany), CD3e (145-2C11; BioLegend, Fell, Germany), pan anti γδ (GL3 BioLegend, Fell) 

### RT-PCR

Small intestine pieces were stored at -80 °C. Tissue was homogenized in RNAmagic (BioBudget, Krefeld, Germany). RNA was extracted and reversely transcribed into cDNA. Quantitative reverse transcription PCR reactions were done with the Rotor-Gene SYBR Green PCR Kit (Qiagen, Hilden, Germany) in a Rotor-GeneQ thermo cycler (Qiagen, Hilden, Germany). The following primers were used: 

GAPDH-F 5'-CGTCCCGTAGACAAAATGGT-3', 

GAPDH-R 5'-TTGATGGCAACAATCTCCAC-3', 

KGF-F 5´-CAAAGGGGTGGAAAGTGAATAC-3´, 

KGF-R 5´-GGAATCCCCTTTTGATTTAAGG-3´; 

FGR2IIIB-F 5´-TGGAGTTTGTCTGCAAGGTTTA-3´; 

FGR2IIIb-R 5´-GTTGGCCTGCCCTATATAATTG-3´; 

GMSF-F 5´-CACAGTCCGTTTCCGGAGTT-3´: 

GMSF-R 5´-GGGTCTACGGGGCAATTTCA-3´; 

IL6-F 5´- TCCAATGCTCTCCTAACAGATAAG-3´, 

IL6-R 5´-CAAGATGAATTGGATGGTCTTG-3´; 

IL10-F 5´-GGTTGCCAAGCCTTATCGGA-3´, 

IL10-R 5´-ACCTGCTCCACTGCCTTGCT-3; 

TGFβ -R 5´-CACTGATACGCCTGAGTG-3´, 

TGFβ -R 5´-GTGAGCGCTGAATCGAAA-3´. 

Expression levels were calibrated to the expression of GAPDH in the same sample using the 2-∆∆CT method (Livak and Schmittgen, 2001[29]).

### Statistical methods

Data were analyzed with GraphPad Prism™ using student´s t-test. Levels of significance are indicated as 

* = P ≤ 0.05, ** = P ≤ 0.01, *** = P ≤ 0.001, **** = P ≤ 0.0001.

## Results

### Changes in intestine-associated immune cells and cytokine profile in AhR-deficient mice

We analyzed the frequency of innate and adaptive T cells, CD103+ DC, and the inflammatory cytokines IL10, IL6, and TGFβ in the small intestine of naïve AhR-deficient mice. In addition we analyzed RNA expression of components of the GM-CSF-pathway. GM-CSF promotes the production of retinoic acid by dendritic cells and expression of FoxP3 in regulatory T cells, thus is a pro-tolerogenic cytokine. The production of GM-CSF in turn is triggered by KGF, a product of γδ T cells (Yokota et al., 2009[45]; Chen et al., 2002[7]). The results are summarized in Table 1(Tab. 1) and 2(Tab. 2). 

As expected from previously published work, the gut γδ T cells were strongly reduced in the AhR-deficient mice. In contrast, lamina propria DCs increased. An increase in the CD103+ subset therein was not significant (P = 0.167). In two independent experiments comparing AhR-deficient mice and their wild-type littermates we did not find differences between the frequencies of DC/CD103+ subset in the mesenteric lymph nodes, nor for CD4+, CD+ FoxP3+ (i.e. regulatory T cells) or CD4+IL17+ (Th17) T cells in the lamina propria or the mesenteric lymph nodes (data not shown). 

Together, the data confirm and extend data showing that in naïve AhR-deficient mice the frequency of intestinal γδ T cells is reduced and DCs are skewed (Li et al., 2011[28]; Kiss et al., 2011[26]). We did not detect differences in genes KGF and the downstream-associated pathway genes Fgr2IIIB or GM-CSF in whole tissue extracts. For GM-CSF we also analyzed supernatants of 4 h *ex vivo* cultivated tissues, but again we did not detect differences between AhR-deficient mice and their WT littermates (data not shown). However, the gene expression of the regulatory cytokine IL10 was significantly reduced in jejunum and ileum of AhR-deficient mice. The pleotropic pro-inflammatory cytokine IL6, which may be involved both in intestinal integrity and in the Treg/Th17 balance (Bettelli et al., 2006[2]), was reduced as well, albeit only slightly and not across the entire intestine (Table 2(Tab. 2)).

### AhR-deficient mice cannot sustain OT

Stability of OT is an important feature of this vital immune function. We therefore tested in preliminary experiments how different feeding protocols affect OT stability upon multiple boosts with the antigen. We immunized up to four times within 47 days after initial OT induction with either 5x0.5 mg OVA, 1x20 mg OVA, or 3x20 mg OVA. Only in the latter dosing scheme, tolerance was sustained, i.e. no increase in antibodies against OVA was detectable in serum after the last immunization. In both the 5x0.5 mg OVA and 1x20 mg OVA protocol anti-OVA antibody levels rose in the serum already upon the second immunization (i.e. first boosting) (data not shown). We therefore used the feeding scheme of 3x20 mg OVA in the experiments whose results are described below. 

**Figure 1:**
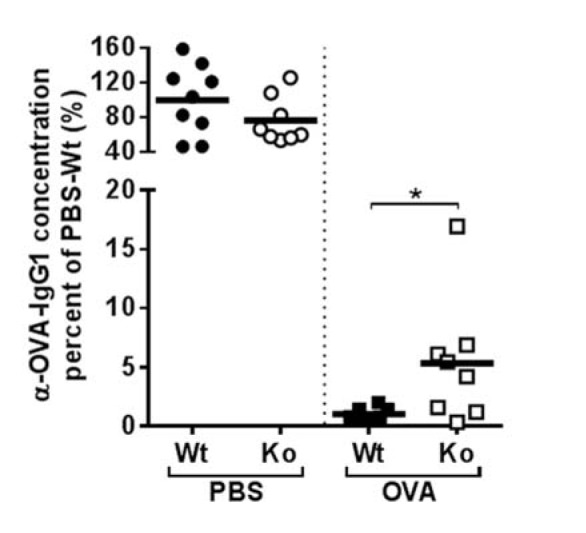
OT stability in AhR-deficient mice. Mice were tolerized orally (3x20 mg OVA i.g.), immunized and then boosted 3 times. One week after the last boost, serum samples were taken and anti-OVA antibodies determined relative to a standard. Black circles or squares: Wild type (Wt) mice, white circles or squares: AhR-deficient (Ko) mice. Each dot depicts the titer from an individual mouse. Data are pooled from three independent experiments. Left side of graph shows results for mice, which had not been tolerized against OVA, but which had been mock gavaged with PBS. Values relative to the mean of the WT PBS group (= 100 %) are shown. *P ≤ 0.05 by student's t-test.

To analyze how AhR-deficiency affects the stability of OT, AhR-deficient mice and their wild-type littermates were tolerized with OVA, then immunized with OVA/CFA and boosted three times with OVA/IFA as described in material and methods. The serum titers of anti-OVA antibodies were determined one week after the last boost, i.e. 47 days after start of the experiment. The result is shown in Figure 1(Fig. 1). 

There was no significant impairment in the capacity to generate a normal antibody response in AhR-deficient mice as such (left side, PBS groups). Wild-type mice sustained OT over three boosts. However, AhR-deficient mice showed a moderate, but significant increase in anti-OVA antibodies in their serum (right side of graph). In other words, tolerance was induced but was not stable after several boosting. We note though, that in three out of eight AhR-deficient mice of the OVA group, no antibodies were produced, i.e. these mice sustained tolerance. 

### Deletion of AhR in T cells, but not intestinal epithelial cells or CD11c+DCs, results in loss of OT

**Figure 2:**
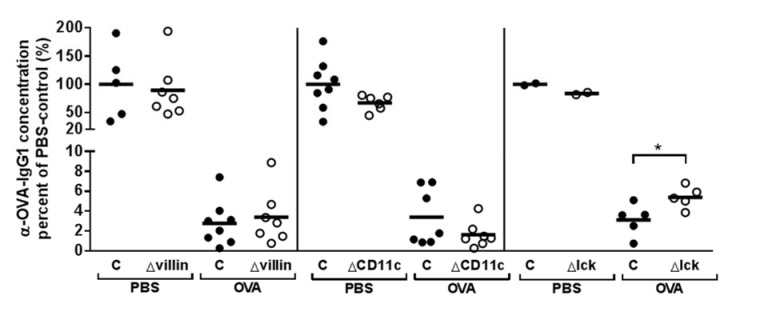
Stability of OT in conditional AhR-deficient mice. Mice deficient for AhR in intestinal epithelial cells (∆villin), dendritic cells (∆CD11c) or T cells (∆lck) were tolerized (3x20 mg OVA), immunized and then boosted 3 times [labeled as OVA in the graph]. As control, mice were fed PBS only [labeled as PBS in the graph]. One week after the last boost, serum samples were taken and anti-OVA antibodies determined relative to a standard. Black circles: Wild-type littermate mice (C), white circles: conditional AhR-deficient mice. Each dot depicts an individual mouse. Values relative to the mean of the respective littermate wild-type PBS group (= 100 %) are shown. *P ≤ 0.05 by student's t-test.

The AhR is abundantly expressed in both the intestinal epithelial cells and several immune cells of the gut (Esser and Rannug, 2015[11]). AhR-expression, or rather the lack thereof, may affect gut barrier integrity, possibly increasing antigen uptake or leakage. Regarding the immune cells, tolerogenicity might be affected, or cytokine production, which may affect the barrier (Ishikawa, 2009[23]; Li et al., 2011[28]; Brandstätter et al., 2016[3]). We therefore ablated AhR cell-specifically in order to identify the cell type relevant for OT destabilization. The results are shown in Figure 2(Fig. 2). 

While OT remained stable if AhR was ablated in either the intestinal epithelial cells or the dendritic cells (Figure 2(Fig. 2) ∆villin and ∆CD11c), it was significantly broken in ∆lck mice. Lck is part of the T cell receptor complex, and it is expressed in all T cells. An exception may be the tissue specific invariant γδ T cells, which develop only in the fetal thymus during short time-windows. These γδ T cells home to their target tissue where they are maintained by homeostatic proliferation. We found that the frequency of γδ T cells in the gut of ∆lck mice was the same as in their wild-type littermates (34.1 % ± 9.6 % vs 32.1 % ± 8.3 %, P = 0.772), and were not lost (as in fully AhR-deficient mice). However, in all conditional mouse lines, anti-OVA titers upon tolerization were still much lower than in the untolerized PBS groups. Note that also in the tolerized wild-type groups, some anti-OVA antibodies were detectable, indicating that even in these mice immunizations led to a minor destabilization of tolerance. 

## Discussion

Food contains numerous proteins (> 100 g protein per day in an average human diet), which are potential antigens and are immunogenic if encountered under immunizing conditions. Yet, we normally do not react to food antigens (Husby et al., 1994[22]; Kapp et al., 2010[25]). It is generally assumed that this immunological unresponsiveness to food antigens results from oral tolerance. Food allergies are highly prevalent, often lead to the so-called atopic march, and their treatment puts a high burden on patients and/or the health system. In this light it is important to better understand OT, as the immunological mechanism that may go wrong in the first place. Also, such knowledge can help inform therapies based on oral desensitization after a food allergy has occurred. Consequently, experimental models of OT in laboratory animals were developed in order to elucidate the underlying mechanisms.

In this paper we provide evidence that the AhR is a player in ensuring continuous oral tolerance against the dietary antigen OVA, even after longer periods of no renewed oral antigen encounter. Second, our data using conditional AhR-deficient mouse lines indicated that primarily AhR expression in CD4+ T cells, but most likely not in gut-associated γδ T cells, is critical for this outcome. 

Models of experimental OT in animals suggested that the dose and feeding regimes can influence which tolerizing mechanism is triggered, i.e. either induction of regulatory T cells or induction of T cell anergy (Friedman and Weiner, 1994[13]; Faria and Weiner, 2005[12]; Weiner et al., 2011[44]). This view has been challenged recently (Pabst and Mowat, 2012[36]), and possibly both mechanisms occur side-by-side in a real-life situation. There may also exist a “no observed effect level” dose, i.e. when food antigens are too rare to necessitate an active OT response. Memory is an intrinsic feature of immune responses and memory T cells are long-lasting. This includes memory Treg cells, whose homeostasis likely is regulated by MHC-II and CTLA-4 contact (Holt et al., 2017[20]). Most models of experimental OT described in the literature look at tolerance induction after one immunization and one boost injection directly following tolerization, and thus do not test for long-term stability of tolerance, i.e. immune memory. One study (Oliveira et al., 2015[35]) tested for persistence of OT by a single feeding of 20 mg OVA i.g., followed with immunizing/boosting 90 days later. In this scenario, the anti-OVA titer was significantly lower than the controls, but present (appr. 50 % of PBS-fed controls), suggesting a slow loss of tolerance over time. Confirming and extending this result, we found that several boosts indeed break tolerance in low- and medium-dose feeding protocols. In contrast, in our model of high-dose feeding (3 x 20 mg i.g.) “tolerance memory” persisted. Only when AhR was absent, tolerance slowly became destabilized and antibodies against OVA detectable. However, the response was weak compared to untolerized mice, i.e., the antibody titers were much lower. It is not easy to say whether there is an antibody threshold for causing or contributing to food allergy caused by a break of oral tolerance. This is a general shortcoming of experimental OT models, as the direct link to food allergy - whether of the IgG or IgE mediated type - is not part of the models. Nonetheless, given that oral desensitization strategies are a method of choice for an existing food allergy, the link is convincing, both theoretically and practically. Of note though, desensitization, i.e. a re-tolerization after an immune response has been established and the damage is done, might entail different mechanisms than the establishment of tolerance upon first antigen encounter. Regarding a correlation between antibody titers and allergy, in human food allergies, even at very low specific IgE levels a food challenge test may be passed, suggesting that even low amounts of antibodies can lead to an allergic reaction to food (Perry et al., 2004[37]). In contrast, in patients with irritable bowel syndrome, no correlation between symptoms and anti-food antigen-specific IgG was detected. The situation may be more complex, as demonstrated by a study in mice by Burton and colleagues (2014[5]). They found that specific IgG antibodies, which were induced upon an oral desensitization protocol, protected from anaphylaxis, even when specific IgE was high. This was mediated by an IgG FcRIIB interaction.

The loss of intraepithelial γδ T cell frequency in the gut is one of the striking findings in the gut-associated lymphoid tissue of AhR-deficient mice (note that such a loss occurs also in the skin (Kadow et al., 2011[24]; Li et al., 2011[28])). γδ T cells are innate immune cells, which are not MHC-restricted like αβ T cells. They have several functions, in particular a first-pass and speedy cytokine secretion upon antigen contact. γδ T cells also secrete factors, which strengthen the tight junctions and intracellular adhesion of the epithelial cells (Vantourout and Hayday, 2013[42]; Lee et al., 2015[27]). Reflecting the decrease in γδ T cell frequency, we detected less RNA for IL10, TGFβ and IL6 in the small intestine of AhR-deficient mice. However, we did not detect changes in expression of genes from the KGF pathway, which supports gut barrier integrity. We think this reflects that γδ T cells are not the major players in our OT model. This may be different for models of food allergy, which use cholera toxin as an “adjuvant” to induce an immune response against the fed peptide antigen. Thus, it was shown recently that gut γδ T cells (but not conventional αβ T cells) take up cholera toxin, and subsequently migrate to the lamina propria, where they secreted IL10 and IL17. The authors conclude that gut γδ T cells may have a major role in food allergy (Frossard et al., 2015[14]). 

Breeding lck-Cre transgenic mice to AhR^flox/flox^ mice, we deleted AhR in T cells. Lck is expressed in all T cells once they begin their differentiation in the thymus. The lck-directed Cre-recombinase expression did not delete AhR in intraepithelial γδ cells. This was entirely unexpected and is currently under further investigation. However, the phenomenon allowed us to distinguish the effect of AhR-ablated conventional T cells from γδ T cells. In the ∆lck mice, OT was impaired upon several immunizations. Also, in ∆lck mice, the γδ T cells were as abundant as in WT littermate controls, in which tolerance did not break. This reinforces our reasoning above that intestinal γδ T cells are not critical in our model of OT stability challenge. Mice with an ablation of AhR in intestinal epithelial cells and DC remained tolerant despite four immunizations. Together this data shows the importance of (conventional) T cells in maintaining oral tolerance. Note though, that we could not detect gross changes in CD4+ and Treg cell subset frequencies in AhR-deficient mice. Obviously, as simple changes in frequencies were inconclusive. Further, and more complex, analyses are needed to identify the respective contribution of T cell subsets.

AhR-deficiency leads not only to loss of γδ T cells in the gut and skin, but also to a failure of innate lymphoid cells ILC3 to develop in the gut (Kiss et al., 2011[26]). The consequences are high bacterial loads in the gut, and susceptibility to the gut pathogen* Citrobacter rodentium*, due to the failure to produce the protective AhR-dependent cytokine IL22 (Kiss et al., 2011[26]; Monteleone et al., 2011[33]). The role of changes in the microbiome by AhR, which might be associated with the changes in bacterial loads, remains to be further explored. Strikingly, it was shown that the removal of AhR-ligands from the diet phenocopied the AhR-deficient mice. Moreover, re-addition of a single AhR-ligand precursor (indole-3-carbinol) into the diet rescued the phenotype (Kiss et al., 2011[26]; Li et al., 2011[28]). The data of these studies made a strong point for the need for a vegetable-containing diet; indeed it provided a molecular mechanism how certain plant compounds such as glucosinolates or flavonoids improve gut health. In similar approaches regarding skin barrier, we also found that an AhR-ligand-deficient diet impairs the skin barrier similar to AhR-deficiency (Haas et al., 2016[17]). We therefore fed an AhR-ligand-deficient diet for three weeks before inducing OT. Unfortunately, using our oral tolerance protocol, we could not mimic the effect of AhR-deficiency (data not shown). While this finding was disappointing, it is possible that non-dietary AhR ligands were present in the mice, e.g. those formed by gut bacteria, or via endogenous generation (Esser and Rannug, 2015[11]). These ligands then would be sufficient for AhR-activity in supporting oral tolerance. In other words, OT may be less sensitive to low levels of dietary ligands than the postnatal generation or proliferation of ILC3 or γδ T cells in the gut. A more trivial explanation is that the feeding had not been long enough. We do not think this is likely, though, as for reduction of γδ T cells in the gut, dietary intervention of three weeks had been sufficient (Li et al., 2011[28]). Even the skin barrier was affected after two weeks of AhR-ligand-deficient diet (personal communication K. Haas).

It is noteworthy in this context, that the antibody titers indicating break of OT were low in absolute terms. Would this matter when considering break of OT as a prerequisite for food allergy? It is well known that the immune system is very flexible and individual variation in many immune parameters can be high. Only once a certain threshold is passed, an adverse outcome ensues. However, we agree with the idea of Hartung and Corsini (2013[18]) who pointed out that the closer an individual is to that threshold of adversity, the easier it is for any added environmental factors (e.g., diet, stress) to push it „over the edge“. Prevention could thus aim at moving the immune system away from the danger zone. In our case, this might be achieved by keeping AhR signaling in the gut in the balance. Some epidemiological evidence, which identified correlations between healthy diet and low allergy risks, underlines this thinking (Grimshaw et al., 2014[15]). 

In conclusion, AhR expression at least in T cells is necessary for stabilizing OT in mice, stressing again the importance of this molecule for mucosal immunity.

## Acknowledgement

We thank Babette Martiensen and Swantje Steinwachs for expert technical help. We thank the staff at our animal facility for their care of the mice. This work was supported by DFG grant ES103/6-1 to C.E. 

## Conflict of interest

The authors declare that they have no conflict of interest.

## Figures and Tables

**Table 1:**
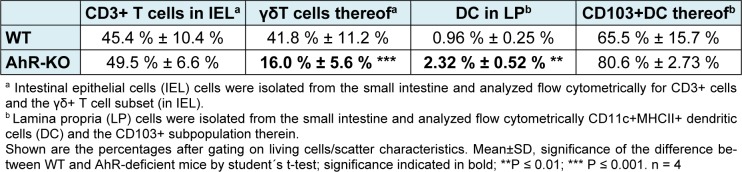
Frequency of T cells and dendritic cells in the intestinal epithelium and lamina propria of AhR-deficient mice and WT littermates

**Table 2:**
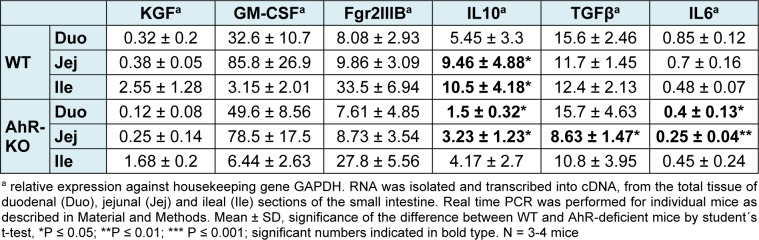
Gene expression in the small intestine
